# Can Planned Renewable Development Mitigate Near‐Term Dry‐Year Electricity Risk in Aotearoa New Zealand?

**DOI:** 10.1002/snz2.70089

**Published:** 2026-10-24

**Authors:** Madison L. Zegeer, Rasul Satymov, Hadi Vatankhah Ghadim, Karan Titus, Jannik Haas, Rebecca Peer

**Affiliations:** ^1^ Sustainable Energy Research Group (SERG) Department of Civil and Environmental Engineering University of Canterbury Ōtautahi Christchurch New Zealand

**Keywords:** capacity expansion planning, dry year, energy security, energy system modelling, hydropower, renewable energy, sustainable energy transition

## Abstract

Dry‐year electricity shortfalls are a defining energy security challenge in Aotearoa New Zealand. Low hydro inflows and ongoing hydro releases deplete seasonal energy storage, increasing reliance on thermal generation, particularly during winter. Amid declining domestic gas reserves and tightening climate commitments, debate persists over how best to maintain system reliability. Our viewpoint provides quantitative modelling evidence, using the open‐source PyPSA‐NZ framework, on whether renewable energy projects in late stages of development can provide reliable electricity during a dry year within the next 5 years. These projects would add 3200 MW of solar photovoltaics, 500 MW of wind power, 130 MW of firm geothermal and 1050 MW of battery storage providing short‐duration flexibility. Because solar and wind are dispatched ahead of hydro, this capacity would displace hydro generation year‐round and raise reservoir storage entering winter. Reliability is secured primarily by this additional stored energy. Using the 2012 hydrological dry year as a stress test, we show that, if these projects are delivered, the modelled system maintains reliable supply without further investment in firm capacity or fuel supply. This suggests the main near‐term risk to electricity reliability is not a shortfall of generation, but constraints on integrating new renewables, including transmission capacity and financing. We propose that near‐term energy security policy prioritises the timely delivery of planned renewable energy projects.

## 1 Energy Security Is a Major Concern in New Zealand's Immediate Future

In winter 2024, Aotearoa New Zealand (NZ) experienced a major electricity crisis. Wholesale prices surged to 800 NZD/MWh for 2 weeks, reflecting a combination of tight hydro conditions, reduced gas supply, and other market and system factors (EA 2025a). As concerns about electricity supply intensified, major industrial users were prompted to reduce their consumption. The 2024 winter reignited a debate about energy security in NZ (WEC 2025), less than a year after the cancellation of the NZ Battery Project (MBIE 2023).

The immediate crisis was mitigated through a combination of reduced electricity use at the New Zealand Aluminium Smelter (NZAS) and the diversion of gas away from industrial consumers. Ultimately, the crisis ended when hydro and wind generation increased later in the year (Schirr 2025). However, the event once again exposed the vulnerability of NZ's electricity system to dry‐year risk. This vulnerability, now coupled with the challenge of declining gas supply (MBIE 2026b), remains a core policy concern with wide‐ranging implications (MBIE 2026a). Several measures to improve NZ's electricity system reliability have been proposed (MBIE 2024b), the most recent being a liquefied natural gas (LNG) import facility, which is in government procurement (MBIE 2026c).

Our viewpoint argues that maintaining electricity reliability during a dry year within the next 5 years will depend on delivering the renewable energy projects already under development, principally new utility‐scale solar photovoltaics (PV) and wind, supported by firm geothermal power and, for short‐duration flexibility, battery storage. Because solar and wind power are dispatched ahead of hydro, this capacity would conserve hydro storage throughout the year. Reliability in a dry year therefore comes from more hydro storage entering winter, not from new generation directly. Rather than developing new modelling methodology, our viewpoint applies an existing, open‐source framework (PyPSA‐NZ) to provide quantitative evidence on an active, publicly contested policy question.

## 2 The Dry‐Year Risk in Context

Hydropower is the backbone of NZ's electricity system, providing ∼60% of annual electricity supply while serving as large‐scale, long‐duration energy storage. NZ hydro reservoir storage is equivalent to approximately 2 months (ranging between 6 and 12 weeks) of annual electricity consumption (MBIE 2023). However, as most hydro storage is concentrated in a small number of South Island catchments, the system is vulnerable to prolonged hydrological deficits (Purdie 2022). NZ's yearly electricity consumption is ∼40 TWh, and a dry year can create a hydroelectricity shortfall of 3–5 TWh (MBIE 2023). Dry years occur when these prolonged periods of low rainfall reduce hydro inflows reduce and reservoir storage. The electricity supply risk is not constant across the year, but most acute towards the end of winter when electricity demand is highest and both inflows and reservoir levels are typically at their lowest (EA 2024a).

NZ has traditionally relied on both coal and gas to offset hydro shortfalls in dry years, with large industrial gas consumers asked to reduce demand and public conservation campaigns targeting households in more severe dry years (Beri and O’Reilly 2017). Declining gas availability intensifies competition for gas between electricity and industrial users and increases reliance on coal (Genesis Energy 2025). NZ operates one 750 MW coal‐fired power station that burns imported coal, exposing the electricity system to international supply chain disruptions and fuel price volatility (MBIE 2025). Recently, the gas outlook has shifted. Methanex, NZ's largest gas consumer and at times using close to half of national supply, has announced its closure and the sale of its gas contracts, releasing a large volume of gas into the market, and Genesis Energy has already secured major contracts through to December 2029 (Ryan 2026). The underlying decline in domestic production nonetheless remains (MBIE 2026c).

Lack of confidence in reliable energy supply may discourage investment in new industries, delay electrification projects and increase costs for energy‐intensive businesses (MBIE 2026a). As a result, dry years not only spark real‐time crises, but also have wider implications for NZ's future economic development (OECD 2025). While considerable attention has been devoted to long‐term (>5 years) solutions (MBIE 2024b; WEC 2025), near‐term reliability depends on what can be delivered within the next few years. New fuel‐supply infrastructure now under procurement could be operational from 2028 (MBIE 2026c). We hypothesise that renewable projects already committed or under development can improve system reliability in the near term.

We base this hypothesis on the scale of recent developments and upcoming projects. More than 900 MW of renewable generation was commissioned during 2024–2025, and 3200 MW of utility‐scale solar PV and 500 MW of wind are scheduled for delivery between 2026 and 2030 (Figure 1) (Transpower 2026b). Since solar and wind rank first in the electricity market merit order (EA 2025b), additional capacity would reduce hydro use throughout the year and increase storage during winter months. Reliability therefore comes from the higher hydro storage this enables.

**FIGURE 1:**
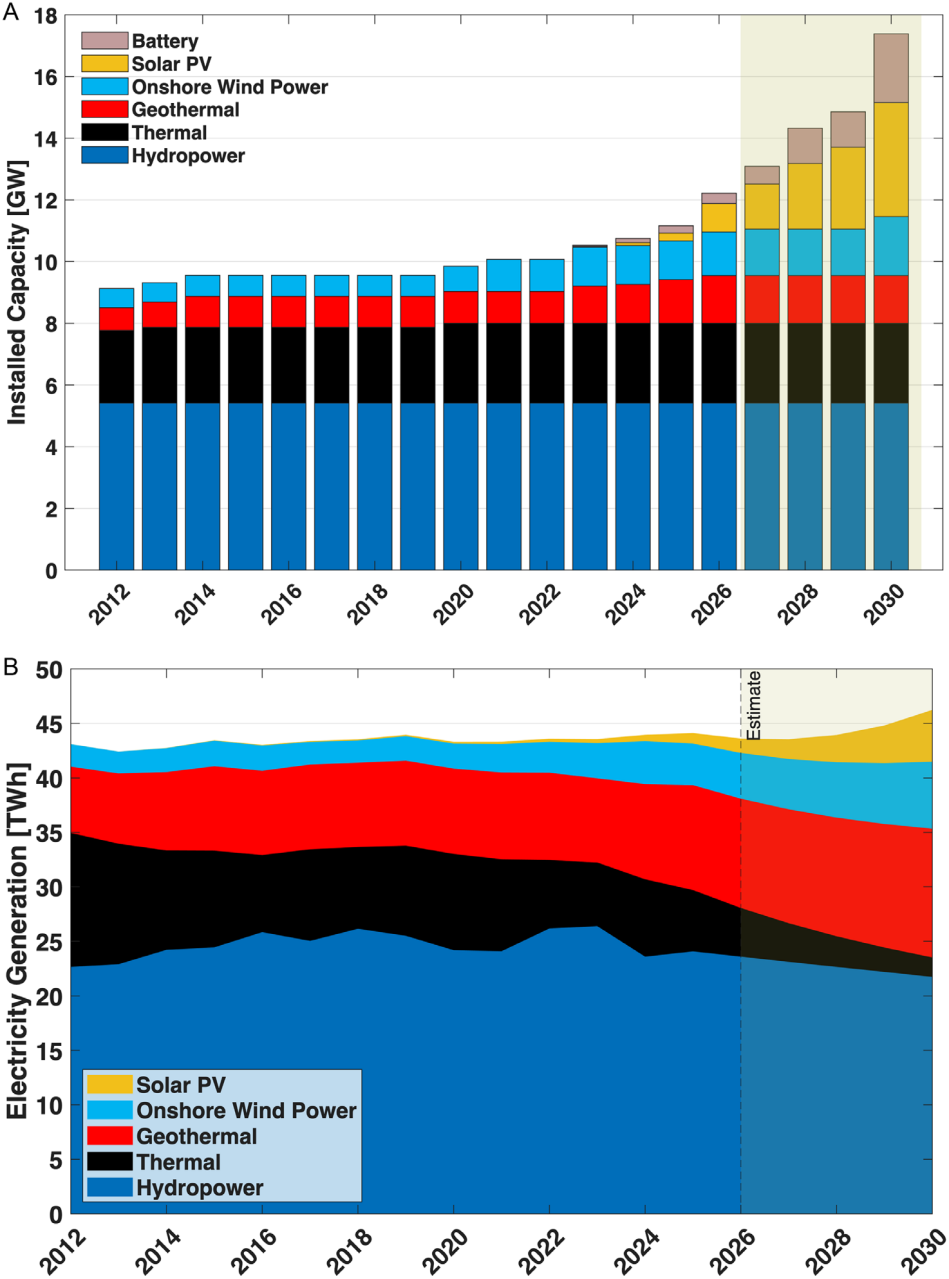
(A) Historical installed electricity generation capacity with expected capacity in the development pipeline by 2030 (shaded region showing planned new projects based on estimated delivery dates). (B) Historical and projected electricity generation beyond 2025 (using an exponential growth trend to interpolate intermediate values between 2025 actual data and 2030 modelling outputs).

## 3 Evaluating NZ's Upcoming Renewable Developments Against a Dry Year

Our viewpoint is underpinned by power systems modelling using PyPSA‐NZ, an open‐source model of the NZ electricity system (Schumm et al. 2026). PyPSA‐NZ solves a linear optimisation problem that determines the least‐cost capacity expansion, generation dispatch, storage management and transmission flows, subject to hourly supply–demand constraints, i.e., 8760 sequential time steps were taken across 16 NZ regions, including periods of peak demand.

### 3.1 Dry Year

To test our hypothesis, we applied a dry year stress test to the PyPSA‐NZ model, defining drought severity as the cumulative deficit in national hydro inflow relative to the long‐term seasonal mean. The 2012 dry year was the most severe drought across the 41 years analysed by Porteous and Mullan (2013), so we emulate its hydrological impacts as a stress‐test scenario for evaluating future dry‐year reliability. We used 2012 hydro inflow values alongside 2012 ERA5 solar irradiation and wind speed data, converting to hourly generation profiles at each site. We selected 2012 over the 2024 event (Section 1) because 2024 reflected a combination of tight hydro conditions, reduced gas supply and market factors rather than an exceptional inflow deficit. Inflow records indicate that 2012 had more sustained inflow shortfalls than 2024 (21.7 TWh vs. 23.6 TWh); therefore, 2012 is a more appropriate scenario for isolating the hydrological mechanisms.

### 3.2 Demand

We scale annual demand to 2030 levels (MBIE 2024a) while retaining the shape of the 2012 hourly load profile (Steijn et al. 2025). However, because our profile retains the shape of 2012 hourly pattern, it does not capture the sharper peak demand that has emerged since the 2023 change to the Transmission Pricing Methodology, which removed the incentive for large users to reduce demand over peaks (Transpower 2022). Our results may understate peak‐period reliability but also do not capture several sources of flexibility that could offset that peak demand. Demand‐side responses, including electric vehicle (EV) smart charging (EVDB 2026), ripple control (EECA 2020) and the NZAS agreement (EA 2024b), can reduce peak demand and diminish the role of gas in the electricity system (Ali et al. 2022; Qadrdan et al. 2017; van Heerden et al. 2025). Rooftop solar PV capacity has also grown rapidly, from 570 MW in 2024–1240 MW by August 2026 (EA 2026), which further reduces grid demand (Zegeer et al. 2025) and increases the value of storage (Cebulla et al. 2018).

### 3.3 Project Pipeline

Table 1 shows our assumptions on the additional renewable generation capacity expected to be developed by 2030. Of the renewable projects scheduled for development between 2026 and 2029 that were included in this comparison, 25 (totalling 2900 MW) had reached the ‘delivery’ stage by September 2026 (Transpower 2026b). We also included 19 projects (2700 MW) in the ‘investigation’ stage with >50% stage completion, which we consider a reasonable threshold for likely deployment by 2030.

**TABLE 1:** Additional capacity indicated by modelling versus advanced‐stage projects in the development pipeline by 2030 (Transpower 2026b).

Technology	Installed capacity, MW Sep 2026	Advance‐stage projects scheduled by 2030, MW	Modelled optimal 2030 installed capacity, MW	Modelled 2030 power output, GWh
Solar PV	640	3200	3840	4800
Wind	1470	500	1970	6100
Geothermal	1420	100	1520	11 800
Hydropower	5200	—	5200	21 800
Gas	1400	—	1400	1100
Coal	750	—	750	600
Batteries	330	1800	2130	—

Under these conditions, PyPSA‐NZ optimises solar PV, onshore wind, geothermal and battery capacity, with expansion capped at pipeline project capacities (Table 1). Hydro and thermal capacities are fixed, and gas and coal availability are unconstrained, so the model can draw on existing thermal plants when needed.

### 3.4 Results

Figure 2 shows electricity supply and demand for the North and South Islands over the year, overlaid with hydro reservoir storage levels. Total hydro storage capacity in the model (∼4500 GWh) reflects dispatchable hydro energy rather than total physical water stored, meaning water below minimum reservoir operating levels is excluded from the modelled storage range from the outset.

**FIGURE 2:**
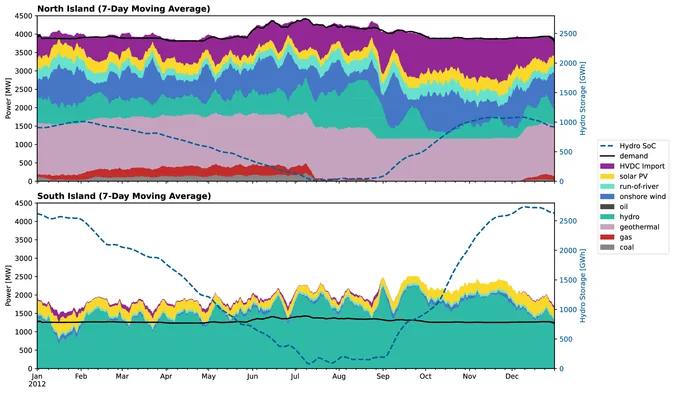
Electricity generation, demand and hydro reservoirs state of charge (SoC) in the 2030 model year, simulated using 2012 dry‐year inflows [HVDC Import – inter‐island transmission link].

In the event of such a dry year, our results indicate that planned, advanced‐stage renewable energy projects are sufficient to meet demand across all 8760 h in the 2030 electricity system. Through optimised dispatch, expanded low‐cost solar PV and wind power reduce gas‐ and coal‐based electricity generation. A further 5300 MW of solar, wind and batteries, plus 400 MW of geothermal (Lawless et al. 2020), are in earlier stages of investigation beyond our modelling pipeline (Transpower 2026b), suggesting that market‐driven renewable energy expansion could continue to mitigate short‐term dry‐year reliability risks beyond 2030, provided planned projects are delivered on schedule.

However, our model assumes hydro generation responds optimally to system‐wide price signals (i.e., conserving water when renewable output is high and releasing it when it is most valuable). Real‐world hydro operators face commercial risk and imperfect foresight that the model does not represent, so actual dispatch may conserve less storage than our results suggest (Conejo et al. 2016). Realising this benefit may therefore also depend on demand response and market mechanisms that better align operators’ incentives with storage conservation.

## 4 Future Outlook and Implications

We show that forthcoming utility‐scale renewable energy projects can offset the reduction in hydropower generation available during a dry year. Prioritising renewables for dry‐year management additionally offers co‐benefits, including lower electricity prices and improved air quality (Galimova et al. 2022). Thus, we propose that the electricity sector's near‐term ability to manage dry years depends on accelerating renewable energy projects.

Recent discussions regarding the construction of an LNG terminal focus on securing fuel supply during future dry years (Firstgas 2026). Considering NZ's climate commitments (Ministry for the Environment 2019; IEA 2023), LNG could reduce emissions if it displaces higher‐emitting fuels such as imported coal. However, net benefits remain contested once upstream emissions from extraction, processing, liquefaction, transport and methane leakage are included in lifecycle assessments (Howarth 2024). Long‐term fuel supply agreements (e.g., take‐or‐pay), typically spanning several decades, may also entrench technological, financial and carbon lock‐in, as long‐lived fossil‐fuel infrastructure creates sunk costs and stranded‐asset risk if replaced early (Seto et al. 2016). Note that these aspects were not explicitly modelled in this study, nor were the potential impacts for industrial gas users outside the electricity sector (Milne 2026).

Under the modelled 2030 demand, project‐delivery, network, technology‐cost and historical dry‐year assumptions, a portfolio dominated by new solar and wind power was sufficient to balance the electricity system without further investment in firm capacity or fuel supply beyond the modelled renewable pipeline.

Our results indicate that NZ has sufficient renewable capacity under development to address near‐term electricity reliability needs, provided planned projects remain on track. Supply constraints are more likely to stem from network limitations and integration hurdles rather than generation shortfalls (CCC 2026). Planned grid upgrades, such as expanding the inter‐island connection from 1.2 to 1.4 GW (Transpower 2026c) and wider use of variable line ratings (Transpower 2026a), may help manage supply–demand mismatches as solar and wind capacities increase, but this is yet to be assessed.

## Funding

This work was supported by the University of Canterbury Sustainable Development Goals Scholarship; the Catalyst: Strategic Fund, administered by the New Zealand Ministry of Business, Innovation and Employment (MBIE) and the German Federal Ministry of Education and Research (grant number 03SF0690), under the project ‘HINT: New Zealand–German Platform for Green Hydrogen Integration’ and the Endeavour Fund Research Programme, administered by New Zealand Ministry of Business, Innovation and Employment (MBIE) (contract number UOC2456), under the project ‘Derisking Carbon Dioxide Removal at Megatonne Scale in Aotearoa'.

## Conflicts of Interest

The authors declare no conflicts of interest.

## Data Availability

Detailed documentation of PyPSA‐NZ is provided in Schumm et al. (2026). Weather‐based energy system inputs were generated using the atlite package (Hofmann et al. 2021), and temporal demand profiles were sourced through the DemandCast package (Steijn et al. 2025). The models used in this study are open source. All other data supporting the findings of this study are included within the article.
